# The spatiotemporal organization of cerebellar network activity resolved by two-photon imaging of multiple single neurons

**DOI:** 10.3389/fncel.2014.00092

**Published:** 2014-04-15

**Authors:** Daniela Gandolfi, Paolo Pozzi, Marialuisa Tognolina, Giuseppe Chirico, Jonathan Mapelli, Egidio D'Angelo

**Affiliations:** ^1^Laboratory of Neurophysiology, Department of Brain and Behavioral Sciences, University of PaviaPavia, Italy; ^2^Laboratory of Experimental and Computational Neurophysiology, Department of Biomedical, Metabolic and Neural Sciences, University of Modena and Reggio EmiliaModena, Italy; ^3^Laboratory of Biophysics and Biophotonics, Department of Physics, University of Milano-BicoccaMilano, Italy; ^4^Laboratory of Neurophysiology, Brain Connectivity Center, C. Mondino National Neurological Institute, Fondazione IRCCS C. MondinoPavia, Italy

**Keywords:** two-photon microscopy, cerebellum, granule cells, Purkinje cells

## Abstract

In order to investigate the spatiotemporal organization of neuronal activity in local microcircuits, techniques allowing the simultaneous recording from multiple single neurons are required. To this end, we implemented an advanced spatial-light modulator two-photon microscope (SLM-2PM). A critical issue for cerebellar theory is the organization of granular layer activity in the cerebellum, which has been predicted by single-cell recordings and computational models. With SLM-2PM, calcium signals could be recorded from different network elements in acute cerebellar slices including granule cells (GrCs), Purkinje cells (PCs) and molecular layer interneurons. By combining WCRs with SLM-2PM, the spike/calcium relationship in GrCs and PCs could be extrapolated toward the detection of single spikes. The SLM-2PM technique made it possible to monitor activity of over tens to hundreds neurons simultaneously. GrC activity depended on the number of spikes in the input mossy fiber bursts. PC and molecular layer interneuron activity paralleled that in the underlying GrC population revealing the spread of activity through the cerebellar cortical network. Moreover, circuit activity was increased by the GABA-A receptor blocker, gabazine, and reduced by the AMPA and NMDA receptor blockers, NBQX and APV. The SLM-2PM analysis of spatiotemporal patterns lent experimental support to the time-window and center-surround organizing principles of the granular layer.

## Introduction

A critical question in understanding brain microcircuit function is the relationship between the properties of single neurons and ensemble network activity. To address this issue it would be desirable to resolve the activity of whole microcircuits with single-cell resolution, but this is still proving difficult. On the one hand the patch-clamp technique allows outstanding resolution of intracellular electrical events (Neher and Sakmann, 1992) but in only few cases has it been shown to be applicable to more than two neurons (e.g., Maffei et al., 2004; Perin et al., 2011). On the other hand, multi-electrode array (MEA) recordings allow mapping of multi-neuronal activity patterns but do not provide information about intracellular membrane potential changes (Nicolelis and Ribeiro, 2002; Buzsáki et al., 2012). Recently, single-or two-photon imaging techniques have made it possible to measure the activity of multiple neurons in brain microcircuits (Yuste, 2005; Peterka et al., 2011; Grienberger and Konnerth, 2012) by adopting scanning heads, acousto-optic deflectors (AODs) (Salomé et al., 2006; Sacconi et al., 2008; Kirkby et al., 2010) or scanless holographic technologies based on spatial-light modulators (SLMs) (Nikolenko et al., 2008). Nonetheless, efficient implementation of these techniques remains critical and their application has been limited.

One microcircuit whose analysis has raised critical issues in physiology is the cerebellar cortex. In particular, the spatiotemporal pattern of activity in the granular layer has, to date, remained elusive. Although Marr's original theory (1969) predicted sparse activation of granule cells (GrCs), 3D-MEA recordings combined with VSD imaging have recently provided evidence of activation in clusters with a center-surround organization (Mapelli and D'Angelo, 2007; Mapelli et al., 2010a,b; Ozden et al., 2012). Moreover, GrC activity in response to mossy fiber bursts consists of just a few (1–3) spikes, and their generation was suggested to be more probable in the center than in the surround, substantiating the time-window hypothesis (D'Angelo and De Zeeuw, 2009; Andreescu et al., 2011; Garrido et al., 2013). These results were supported by mathematical modeling (Solinas et al., 2010; Diwakar et al., 2011) suggesting a precise distribution of active neurons in the network, although no direct experimental demonstration was provided. Similarly, activity in multiple Purkinje cells (PCs) has been shown using electrophysiological and imaging techniques (Sacconi et al., 2008), but there are, as yet, no reports on the relationship between activity in PCs and other granular layer and molecular layer neurons.

To address this aspect, we implemented an advanced two-photon laser microscope employing a spatial-light modulator (SLM-2PM) to elicit fluorescent responses in selected neurons in acute cerebellar slices (Nikolenko et al., 2008). This scanless system allowed the generation of arbitrary holographic patterns of excitation providing stable recordings of multiple neurons with high spatiotemporal resolution (1–2 kHz sampling). Fura-2 calcium signals could be recorded from all the different network elements, thus allowing, for the first time, monitoring of synaptic responses in tens to hundreds of GrCs, PCs and molecular layer interneurons (MLIs) simultaneously, and supporting, at the single-cell level, the time-window and center-surround hypotheses regarding the organization of the granular layer.

## Methods

In order to obtain arbitrarily defined multi-site recordings from neurons of the cerebellar network, we implemented an advanced imaging system (SLM-2PM) (Nikolenko et al., 2008) based on fast (up to 2 kHz) calcium imaging (Fura-2).

### The SLM-2PM

**Figure 1:**
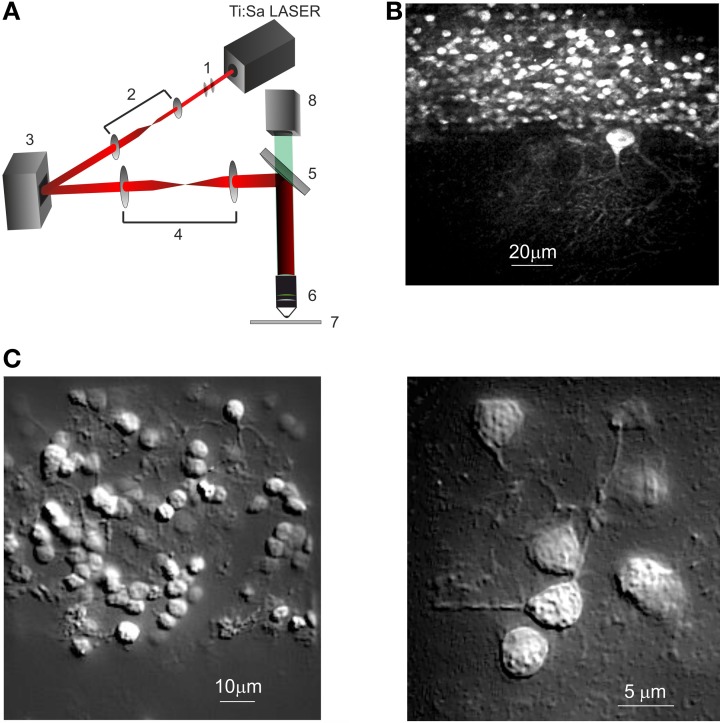
**Spatial light modulator—two-photon microscope (SLM-2PM). (A)** Schematic representation of the SLM-2P Moptical system. (1) Polarizer and half-wave plate, (2) beam expander, (3) SLM, (4) beam reducer, (5) dichroic mirror, (6) objective, (7) specimen, (8) CMOS camera. **(B)** SLM-2PM image of a cerebellar slice bulk-loaded with Fura-2AM, illuminated with *sliding grid scan* and reconstructed at low magnification (20× objective). Several GrCs and one PC with a large dendritic tree are visible. **(C)** SLM-2PM images processed offline to show neuronal details. A portion of the granular layer (*left*, 40× objective) shows numerous GrCs and their neuritic prolongations. Three GrCs (*right*, 60× objective) send their dendrites toward a common point, presumably corresponding to a mossy fiber terminal.

Our imaging system consists of a custom-made upright microscope that employs a diffractive spatial light modulator (SLM) to produce any desired spatial profile of excitation light on the sample plane (Figure 1A). We use an LCOS-SLM optical phase modulator (X10468-07, Hamamatsu, Japan), which has a resolution of 800 × 600 pixels, 8-bit phase quantization, and, for visible and near infrared light, is capable of complete 2π phase modulation at each pixel, with a 60-Hz refresh rate. The SLM takes advantage of Fourier optics to perform computer-driven holographic microscopy, by spatially modulating the phase of a coherent light source. The phase distribution (phase mask) which generates the desired illumination pattern was computed with custom-developed routines written in Python (PSF, 9450 SW Gemini Dr. Beaverton, OR 97008, USA) based on standard iterative-adaptive algorithms (Gerchberg, 1972; Nikolenko et al., 2008).

The illumination path begins with the two-photon light source, a mode-locked ultrafast Nd:YVO4 pumped Ti:Sa laser that generates femtosecond pulses at a repetition rate of 80 MHz (Coherent Chameleon Ultra II, California) (Figure 1A). A polarizer is placed along the optical path since the LCOS-SLM is sensitive to the polarization of the incident light. The beam power is regulated by means of a half-wave plate. The beam passes through a telescopic beam expander (*f*_1_ = 2.5 cm, *f*_2_ = 15 cm, Thorlabs Inc. Newton, NJ, USA), used to optimally fill the SLM active surface. The light modulated by the SLM surface passes through a telescopic beam reducer (*f*_3_ = 40 cm, *f*_4_ = 25 cm, Thorlabs Inc. Newton, NJ, USA), which reduces the wave-front diameter to fill the objective back-aperture. Finally the light is directed onto the sample through the objective by a dichroic filter that reflects wavelengths longer than 750 nm. All the microscope optics were fixed onto a vertical honeycomb steel breadboard and bright-field Köhler illumination was used for sample illumination. The two-photon fluorescence for image generation was collected with a high spatial resolution CCD camera (CoolSnap, Photometrics, Tucson, USA), and processed with MetaMorph (Version 6.1, Molecular Devices) and ImageJ (NIH, Bethesda, Maryland, USA) while calcium signals generated in response to neuronal network activation were collected through an ultra-fast CMOS camera (MICAM Ultima, Scimedia, Japan).

A small fraction (<25%) of the incoming light remains undiffracted (the “zero-order” beam). We currently use an “on-center” configuration wherein the undiffracted beam is present in the field of view, and we employed a small beam-stop to remove it. Imaging was done at depths of 50–60 μm, using water-immersion objectives with different magnification (Olympus LUMPlanFl 60× 0.9 N.A.; Zeiss Plan-APOCHROMAT 20× 1.0 N.A.; Zeiss Plan-APOCHROMAT 40× 1.0 N.A.).

### Image generation and cell identification

The SLM-2PM, by using a SLM to shape an incoming laser beam into any arbitrary light pattern, was an effective scan-less microscope. The two-photon fluorescence images, used to identify cell bodies positions, were indeed generated without any moving parts (e.g., conventional raster scanning with galvanometric mirrors). SLM phase masks are computed with custom developed software based on FFT in classic Gerchberg-Saxton algorithm which, given a 600 × 600 pixel grayscale image of the desired illumination pattern, calculates the corresponding 800 × 600 pixel phase mask. In order to generate images of the sample, an SLM phase mask is computed in order to split the laser beam in a regular grid of diffraction-limited beamlets (*sliding grid*). This *sliding grid scan* procedure was employed in two different modes: (i) *live imaging* mode, showing low resolution confocal images directly on the camera output; (ii) *high resolution* mode, producing digital confocal images, useful for the selection of points of interest for signal acquisition.

#### Live imaging mode

Two-photon live imaging was obtained through the projection of the *sliding grid* at the maximum SLM refresh frequency (60 Hz). The CoolSnap camera acquired the image in a single exposure lasting for the entire *sliding grid* sequence (600 ms). Using a *sliding grid* of 28 × 28 points moving in a 6 × 6 raster-scan pattern, the live stream of images occurred at 1.66 frames per second. The scanning sequence covered a 110 × 150 μm^2^ rectangular area (40× magnification; the rectangular shape was due to the application of FFT on rectangular matrices).

#### High resolution mode

High resolution two-photon imaging was achieved with a *sliding grid* of 30 × 30 points, moving in a 20 × 20 raster-scan pattern to cover the same field of view of the *live imaging mode*. In this case, the CoolSnap camera exposure corresponded to each phase mask projection, resulting in a sequence of 400 separate images. The minimum time required for image acquisition in this configuration with a 60 Hz SLM refresh rate was around 7 s. Depending on signal intensity, longer exposures were used to improve the S/N ratio. In a standard configuration, using 225 ms exposure, 400 images were collected in about 90 s. The stack of the 400 images was elaborated by using custom software implementing the following steps: (i) computing the expected position of each beamlet of the scan sequence on the CCD images; (ii) recovering the fluorescence signal coming from each beamlet position in every frame. (iii) obtaining a 600 × 600 pixel grayscale image, in which every pixel value is proportional to the fluorescence signal generated by the corresponding beamlet. The images obtained with the *high resolution mode* exactly matched the input of the Gerchberg-Saxton algorithm, which was used to generate arbitrary illumination patterns pointing to specific neuronal structures in the sample (such as cell bodies or dendrites) with sub-micrometer precision (*multi-spot illumination*).

The system resolution has been tested experimentally with 0.1 μm fluorescent beads in agarose gel using a Zeiss Plan-APOCHROMAT 40× 1.0 N.A. objective, resulting in 0.4 μm lateral resolution and 1.5 μm axial resolution.

### Slice preparation and electrophysiology

Acute cerebellar slices (200 μm thick) were obtained from 18- to 25-day-old Wistar rats. Rats were anesthetized with halothane (0.5 ml in 2 L for 1–2 min) before being killed by decapitation. All experiments were conducted in accordance with international guidelines on the ethical use of animals (European Community Council Directive 86/609/EEC). The cerebellum was gently removed, and the vermis was isolated, fixed with cyanoacrylate glue, and immersed in cold (2–3°C) cutting solution. Slices were cut in the sagittal plane. The cutting solution contained (in mM): 130 K-gluconate, 15 KCl, 0.2 EGTA, 20 HEPES, 10 glucose; the solution was brought to pH 7.4 with NaOH. Slices were then transferred to an oxygenated Krebs' solution containing (in mM): 120 NaCl, 2 KCl, 1.2 MgSO_4_, 26 NaHCO_3_, 1.2 KH_2_PO_4_, 2 CaCl_2_, 11 glucose; pH 7.4 when equilibrated with 95% O_2_–5% CO_2_. For extracellular bulk loading, slices were deposited on the bottom of a small Petri dish (35 mm × 10 mm) filled with 2ml of ACSF, ventilated with 95%O_2_/5%CO_2_ and placed in a warmer (PBI International) at 34°C. A 50 μ g aliquot of Fura-2AM (Molecular Probes, Eugene, OR, USA) was prepared in 48 μ l DMSO and 2 μ l Pluronic F-127 (Molecular Probes). The dye aliquot was then placed on top of the slice in the Petri dish and slices were incubated in the dark at 35–37°C for up to 60 min. Slices were maintained at room temperature for at least 30 min before being transferred to the recording chamber. Slices were gently positioned in the recording chamber and fixed with a nylon mesh attached to platinum Ω-wire to improve tissue adhesion and mechanical stability. Perfusion of oxygenated Krebs' solution at room temperature was continued during the recording session.

Current-clamp recordings (WCRs) were made in whole-cell patch-clamp configuration from GrCs as reported previously (D'Angelo et al., 1995). Recordings were obtained with an Axopatch200A amplifier (Molecular Devices, Union City, CA,USA) (3-dB cut-off frequency 10 kHz) and data were digitized at 20 kHz with a Digidata 1322A A/D converter (Molecular Devices) using pClamp9 software (Molecular Devices). Patch pipettes were pulled from borosilicate glass capillaries (Hilgenberg, Malsfeld, Germany) and filled with the following solution (in mM): 126 K-gluconate, 4 NaCl, 15 glucose, 5 HEPES, 1 MgSO4^*^7 H2O, 0.1 BAPTA-free, 3 ATP, 100 μm GTP; pH was adjusted to 7.2 with KOH. This solution maintained resting free [Ca^2+^] at 100 nM. Intracellular solution was added with cell membrane impermanent calcium dye, 200 μ M Fura-2.

For GrC recordings, the pipettes had a resistance of 7–10 MΩ before seal formation. The stability of patch-clamp recordings can be influenced by modifying series resistance and neurotransmitter release. To ensure that series resistance remained stable during the recordings, passive cellular parameters were extracted in voltage-clamp mode by analyzing current relaxation induced by a 10 mV step from a holding potential of −70 mV. According to previous reports (D'Angelo et al., 1995), the transients were reliably fitted with a mono-exponential function yielding membrane capacitance (*C*m) of 4.2 ± 0.1 pF (*n* = 5), membrane resistance (*Rm*) of 1.9 ± 0.3 GΩ (*n* = 5) and series resistance (*Rs*) of 19.4 ± 0.7 MΩ (*n* = 5). The −3 dB cell-electrode cut-off frequency, *f*VCD = (2π*R*_s_*C*_m_)^−1^, did not significantly change during recordings. Mossy fibers were stimulated with a bipolar tungsten electrode (Clark Instruments, Pangbourne, UK) via a stimulus isolation unit and stimulation intensity (4 ± 8 V; 100 μs) was raised until the EPSPs generated spikes from a membrane potential between −70 and −60 mV (mean −63.7 ± 4.7; *n* = 5). On the basis of previous data (D'Angelo et al., 1995; Sola et al., 2004), between one and three mossy fibers were stimulated per GrC.

Recordings from PCs were performed as previously reported (Sacconi et al., 2008; Mapelli et al., 2010a). Briefly, WCRs were performed from PCs using pipettes containing the same intracellular solution employed for GrCs. With this solution, the pipette resistance was 3–4 MΩ. All PCs showed spontaneous firing in cell-attached configuration (13.1 ± 4.9 Hz, *n* = 4) as well as after passing into the whole-cell recording configuration in current clamp mode (14.8 ± 3.0 Hz, *n* = 4). The stimulation of the white matter in the granular layer generated EPSPs, IPSPs as well as simple and complex spikes depending on the stimulation intensity and on the position of the stimulating electrode (data not shown). Passive properties of PCs, estimated from current relaxation during a voltage step from −65 to −70 mV in a way similar to that adopted for GrCs, yielded membrane capacitance (*C*m) of 612 ± 20.4 pF (*n* = 6), membrane resistance (*R*m) of 64.6 ± 2.5 MΩ (*n* = 6) and series resistance (*R*s) of 13.4 ± 0.5 MΩ (*n* = 6). Data used for correlating intracellular membrane depolarization with the calcium signals were taken after preventing spontaneous firing with negative current injection (≤ −200 pA).

### Data analysis

Intracellular calcium increases generated Fura-2 fluorescence decreases which were collected at variable sampling frequency (from 0.02 to 2 kHz) and presented as fluorescence variations normalized with respect to the initial background fluorescence (ΔF/F_0_). Signal analysis was performed by evaluating the following parameters: (i) peak amplitude (difference between signal peak and baseline), (ii) time-to-peak (ttp: time delay between the stimulus and the peak of the response); (iii) latency (time delay between the stimulus and the time required to reach 20% the peak response); (iv) rising phase (difference between ttp and latency); (v) ΔF/F_0_ area (integral of the ΔF/F_0_ signal). Given a peak amplitude of 3–5% and a noise standard error of about 0.5% (σ = 0.41 ± 0.02%), the ΔF/F_0_ signal-to-noise (S/N) ratio was increased about 10-fold ensuring a reliable measurement of peak response amplitude. The number of active neurons was determined by considering responses larger than 2σ (1% ΔF/F_0_). In combined WCR and imaging experiments, electrophysiological traces were analyzed by counting the number of emitted spikes following synaptic activation and by measuring the total depolarizing area generated by EPSPs in sub-threshold responses. The center-surround organization was mapped using the excitatory/inhibitory balance, *E*/*I* = (*E*_norm_ − *I*_norm_)/*E*_norm_. In this map, *E*_norm_ is the response intensity normalized to the maximum response and *I*_norm_ is the response variation following gabazine perfusion normalized to the maximum, so that the map values range from 1 (maximal excitation) to −1 (maximal inhibition) (cf. Mapelli and D'Angelo, 2007). Stack images were obtained by normalizing and centering each experiment on the cell showing maximum response in control condition. The stack images were filtered with a sliding box (average value; 3 × 3 pixels).

Data are reported as mean ± standard error of the mean (s.e.m).

## Results

In the cerebellar cortex, mossy fiber stimulation excites GrCs, whose spikes travel along the ascending axon and parallel fibers activating PCs and MLIs. This sequential activation has previously been demonstrated in brain slices using both electrophysiological and imaging recordings (Mapelli et al., 2010a,b).

The activity of the cerebellar microcircuit was investigated by recording calcium signals from multiple single neurons with SML-2PM (Figure 1A) in acute slice preparations (P18-P25) (see Methods). Following bulk loading of slices with Fura-2AM, numerous cells became fluorescent (Figure 1B) and were identified as neurons based on their morphology and neuritic processes (Figure 1C).

**Figure 2:**
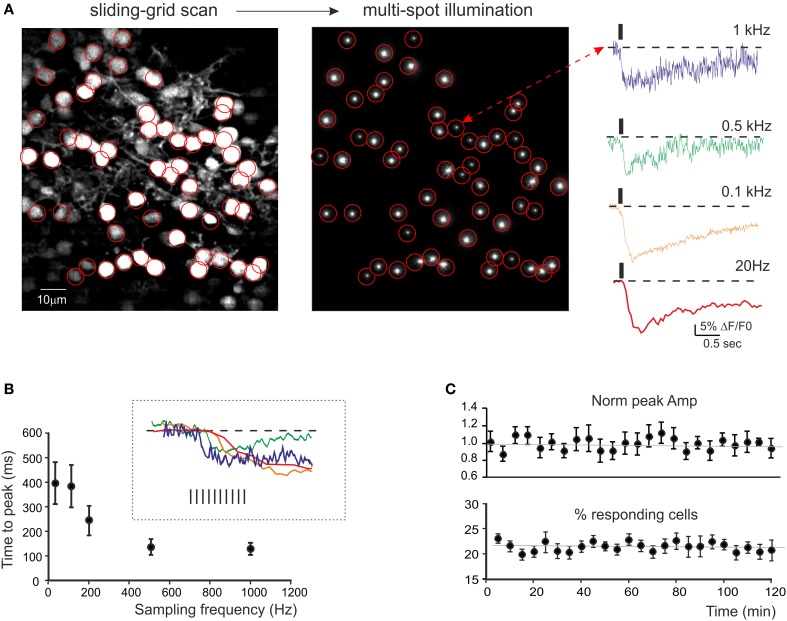
**Calcium signals from cerebellar GrCs. (A)** SLM-2PM image generated by Fura-2AM bulk loading of a cerebellar slice illuminated with *sliding grid scan* and reconstructed on-line. *Multi-spot illumination* was generated by pointing the laser beamlets toward selected neurons. The Ca^2+^ signal variations detected from the soma of GrCs in response to mossy fiber stimulation (10 impulses at 50 Hz) appear as a ΔF/F_0_ reduction induced by intracellular calcium increase in GrCs. The traces on the right show the time course of ΔF/F_0_ at different sampling frequencies (up to 1 kHz) taken from a representative GrC. It should be noted that calcium signals show faster kinetics by raising the sampling frequency. **(B)** The plot shows the relationship between time-to-peak and sampling frequency (average from 21 GrCs in a single experiment, mean ± s.e.m.). The inset shows the rising phase of the traces in **(A)**. The time-to-peak becomes faster and rising sampling frequency tends to plateau around 0.5 kHz. **(C)** Time course of ΔF/F_0_ measured at the peak of calcium signals elicited by mossy fiber burst stimulation (10 impulses at 50 Hz) at 0.1 Hz (average from 54 GrCs in three experiments, mean ± s.e.m.) and of the average number of active GrCs. Note signal stability over 2 h of recordings.

Mossy fiber stimulation caused fluorescence reduction in some neurons, indicating a corresponding intracellular calcium increase (Figure 2A, see Movie in Supplemental Material). The neurons to be used for recording were selected by drawing specific regions of interest (ROIs) through the *multi-spot illumination* procedure (see Methods) and the stimulus-induced fluorescence changes, ΔF/F_0_, were analyzed offline. The initial fluorescence intensity F_0_ was obtained from the average baseline signal valueand ΔF/F_0_ was then calculated for each subsequent frame. Signals were sampled at different frequencies (0.02–2 kHz) in order to evaluate the S/N ratio and the time-course of ΔF/F_0_(Figure 2A). In GrCs, at 0.5–1 KHz, ΔF/F_0_ peaked in about 100 ms (140.5 ± 33.5 ms at 500 Hz and 133.6 ± 24.8 at 1 kHz, *n* = 10 experiments) with values of 5–10% (7.2 ± 0.6%, *n* = 10 experiments) and a S/N ratio of 11.4.

Signal stability was monitored by repetitively stimulating the mossy fibers at 0.005 Hz and measuring the ΔF/F_0_ and the number of responsive cells. In GrCs, these two parameters showed remarkable stability over 2 h (−0.1 and −1% decrease, respectively) (Figure 2C). This probably reflected the low light intensity (3–5 mW/μm^2^) and the small illuminated area, which could limit the production of toxic metabolites and photo-bleaching (see also Salomé et al., 2006; Sacconi et al., 2008; Dombeck et al., 2010).

**Figure 3:**
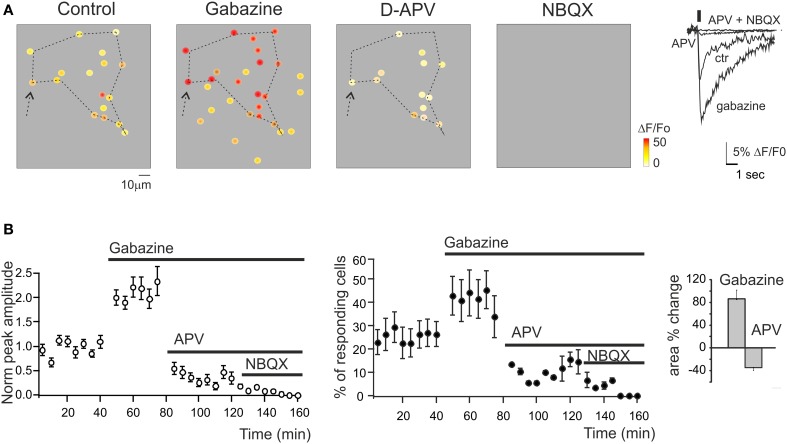
**Regulation of GrC activity by synaptic receptor blockers. (A)** Activity maps of the granular layer showing the peak intensity of neuronal responses on a color scale. The location of colored dots corresponds to that of neurons in the slice. The four panels (from left to right) show that both the activation intensity and the number of active GrCs increase remarkably during 10 μ M gabazine application, decrease following addition of 50 μ MAPV, and are fully blocked following further addition of 20 μ M NBQX. Note the variation of the responding area (dashed lines) following the application of gabazine and APV (+73.1 and −24.5% compared to control conditions). The traces on the right show the ΔF/F_0_ changes caused by the drugs in a selected GrC (arrow). (**B)** Time course of the normalized peak amplitude and of the number of responding GrCs in different experiments following the application of synaptic blockers (*n* = 254 cells; *n* = 15 slices; mean ± s.e.m.). In all these experiments, ΔF/F_0_ was measured at the peak of Fura-2AM calcium signals elicited by mossy fiber burst stimulation (10 impulses at 50 Hz) at 0.1 Hz.

**Figure 4:**
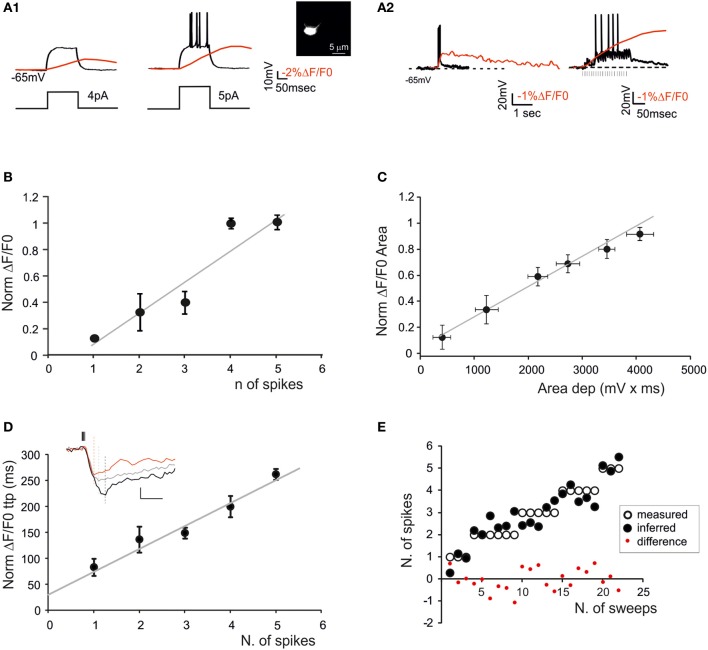
**The relationship between calcium signals and GrCactivity**. Fura-2 was injected intracellularly in GrCs through the patch pipette (inset) and electrical and SLM-2PM calcium signals were recorded simultaneously. **(A1)** Voltage traces showing responses of a GrC to a 30-ms step-current injection (left 4 pA; right 5 pA). Note that the transition from the sub-threshold (left) to the spiking regime (right) is correlated with an increase in the calcium transient (red traces). **(A2)** Voltage traces showing EPSP-spike complexes induced by a mossy fiber burst (20 stimuli at 100 Hz). Note the sustained calcium transient correlated with action potentials. **(B)** Dependence of normalized ΔF/F_0_ peak on the number of spikes emitted by GrCs in response to mossy fiber bursts (20 stimuli at 100 Hz) revealing a linear correlation (gray line; *n* = 5 cells *R*^2^ = 0.85; *p* < 0.05; mean ± s.e.m.). **(C)** Dependence of normalized ΔF/F_0_ area on the average depolarization in response to mossy fiber bursts (20 stimuli at 100 Hz) revealing a linear correlation (gray line *n* = 5 cells; *R*^2^ = 0.98; *p* < 0.001; mean ± s.e.m.). Only sub-threshold responses were considered. **(D)** Dependence of normalized ΔF/F_0_ time-to-peak on the number of spikes emitted in response to mossy fiber bursts (20 stimuli at 100 Hz) revealing a linear correlation (gray line; *n* = 5 cells; *R*^2^ = 0.94; *p* < 0.01; mean ± s.e.m.). The traces in the inset show ΔF/F_0_ signals correlated with 1 (red), 3 (gray), and 5 spikes (black) **(E)** Comparison of the number of spikes measured in GrCs and that predicted by the slope of the time-to-peak *vs*. spike relationship shown in **(D)**. Note the matching between predicted and measured results (two-tailed *t*-test; *p* = 0.82).

**Figure 5:**
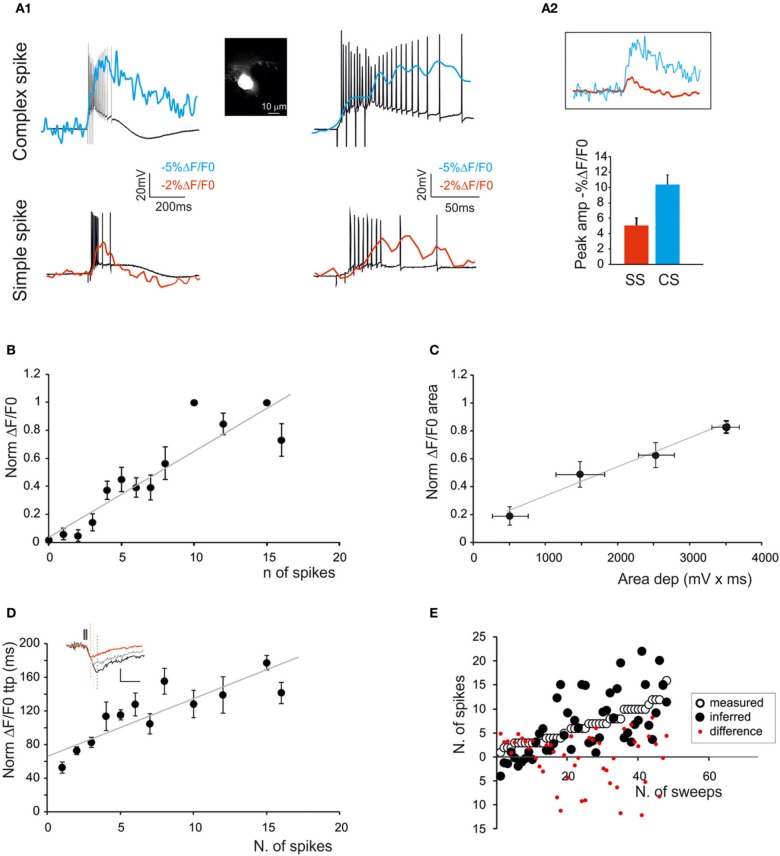
**The relationship between calcium signals and PC activity**. Fura-2 was injected intracellularly in PCs through a patch pipette (inset; scale bar 10 μm) and electrical and SLM-2PM calcium signals were recorded simultaneously. **(A1)** Voltage traces showing EPSP-spike complexes induced by a mossy fiber burst (5 stimuli at 100 Hz). Note the sustained depolarization induced by the presence of action potentials. Simple spikes (bottom panels) and complex spikes (upper panels) generate different calcium signals. **(A2)** The calcium signals generated by simple and complex spikes are compared. The histogram shows larger size of complex-than simple-spike calcium signals. **(B)** Dependence of normalized ΔF/F_0_ peak on the number of simple spikes emitted by PCs in response to mossy fiber bursts (5 stimuli at 100 Hz) revealing a linear correlation (gray line; *n* = 4 cells; *R*^2^ = 0.9; *p* < 0.01; mean ± s.e.m.). **(C)** Dependence of normalized ΔF/F_0_ area on the average depolarization during simple spike discharge in response to mossy fiber bursts (5 stimuli at 100 Hz) revealing a linear correlation (gray line; *n* = 4 cells *R*^2^ = 0.63; *p* < 0.05; mean ± s.e.m.). Only sub-threshold responses were considered. **(D)** Dependence of normalized ΔF/F_0_ time-to-peak on the number of simple spikes emitted in response to mossy fiber bursts (5 stimuli at 100 Hz) revealing a linear correlation (gray line; *R*^2^ = 0.56; *p* < 0.01; mean ± s.e.m.). The traces in the inset show ΔF/F_0_ signals correlated with one (red), five (gray) and 10 spikes (black) **(E)** Comparison of the number of spikes actually measured in PCs and that predicted by the slope of the time-to-peak vs. spike relationship shown in **(D)**. Note the matching between predicted and measured results (two-tailed *t*-test; *p* = 0.99).

**Figure 6:**
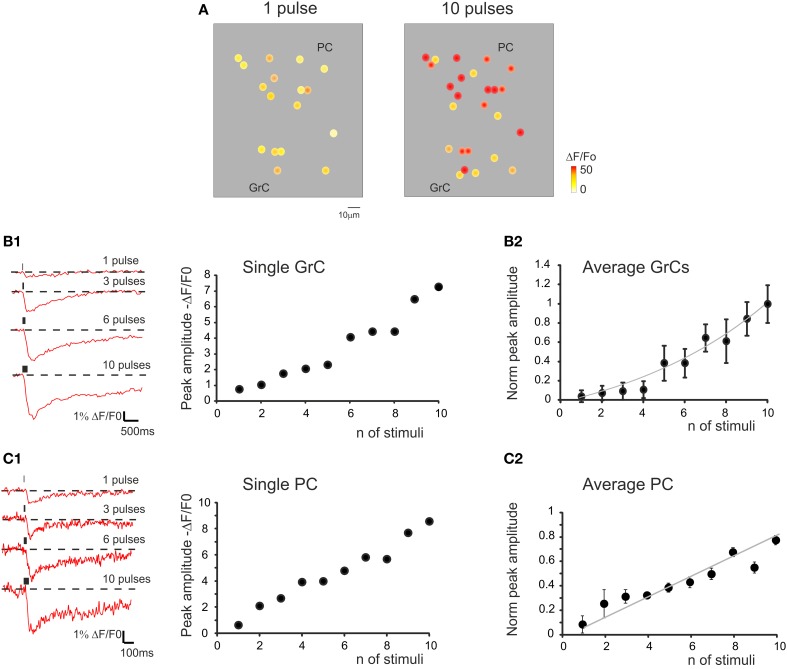
**The relationship between calcium signals and mossy fiber activity. (A)** Activity maps of a cerebellar slice showing the peak intensity of neuronal responses on a color scale. The location of colored dots corresponds to that of neurons in the slice. The two panels (from left to right) show that both the response intensity and the number of active GrCs increase remarkably by raising the number of stimulation pulses. **(B1)** Traces on the left illustrate the time course of ΔF/F_0_ obtained from a GrC in response to an increasing number of mossy fiber stimuli at 50 Hz. The plot on the right shows the values of ΔF/F_0_. Note the increase in normalized peak amplitude from one to ten stimuli. **(B2)** The plot shows the average values of ΔF/F_0_. Note the increase in normalized peak amplitude from one to ten stimuli (*n* = 35 cells in six experiments; exponential fitting *R*^2^ = 0.94; *p* < 0.01; mean ± s.e.m.). **(C1)** Traces on the left illustrate the time course of ΔF/F_0_ obtained from a PC in response to an increasing number of mossy fiber stimuli at 50 Hz. The plot on the right shows the values of ΔF/F_0_. Note the increase in normalized peak amplitude from one to ten stimuli. **(C2)** The plot shows the average values of ΔF/F_0_. Note the increase in normalized peak amplitude from one to ten stimuli (*n* = 13 cells in five experiments; linear fitting *R*^2^ = 0.81; *p* < 0.05; mean ± s.e.m.). In all these experiments, ΔF/F_0_ was measured at the peak of Fura-2AM calcium signals elicited by mossy fiber stimulation.

The neuronal origin of ΔF/F_0_ signals was determined not only by (i) generating high-resolution confocal images allowing visual matching between fluorescent signals and neuronal elements (see Figure 1 and Supplemental Material) and (ii) stimulating the afferent mossy-fiber bundle to elicit fluorescence changes through synaptic activation, but also by (iii) evaluating the effect of synaptic receptor antagonists (Figures 2, 3), (iv) correlating ΔF/F_0_ with the electrical response in WCRs (Figures 4, 5), (v) correlating ΔF/F_0_ values with the mossy fiber stimulus patterns (Figure 6), and (vi) reconstructing cerebellar network activation (Figures 8, 9).

### The synaptic-dependent nature of network responses: synaptic receptor blockers

The synaptic-dependent nature of the responses was directly assessed by applying blockers for ionotropic GABA and glutamate receptors (Figure 3). GrCs express several GABA-A receptor subunits (Brickley et al., 1999; Farrant and Nusser, 2005). Blockade of GABA-A receptor-mediated synaptic inhibition is known to increase spike discharge and to depolarize the GrCs, increasing transmembrane Ca^2+^ influx (Martina et al., 1994). In SLM-2PM recordings, the perfusion of a solution containing the GABA-A receptor blocker, 20 μ M gabazine, increased both the peak amplitude (+129.9 ± 2.5%; *n* = 15 slices; *p* < 0.001) and the number of active cells (+89.1 ± 11.3%; *n* = 15 slices; *p* < 0.01). After 30 min washing with control Krebs' solution, the calcium signals recovered close to the initial conditions (−3.5 ± 1.8% *n* = 5, *p* > 0.65). Gabazine application was followed by coperfusion of glutamate receptor blockers. GrCs show a remarkable expression of NMDA receptors (Garthwaite and Brodbelt, 1989; D'Angelo et al., 1990) (Figure 3). Blockade of NMDA receptors is known to markedly decrease EPSP temporal summation and spike discharge, mostly in response to high-frequency mossy fiber bursts. In SLM-2PM recordings, perfusion of a solution containing the NMDA receptor blocker, 50 μ M APV, markedly reduced both the peak amplitude (−75.5 ± 12.3% *n* = 6, *p* < 0.01) and the number of active cells (−79.6 ± 9.3% *n* = 6, *p* < 0.01 Figure 3). The residual GrC activity was completely blocked by application of the AMPA receptor blocker, 10 μM NBQX (−100% *n* = 5, *p* < 0.01). It should also be noted that the area covered by responding GrCs increased with gabazine (+86.3 ± 15.5% *n* = 6; *p* < 0.01) and decreased with APV (−34.9 ± 4.4% *n* = 6; *p* < 0.01), according to the lateral inhibition and center-surround organization reported in the granular layer (Mapelli and D'Angelo, 2007).

### Correlation of calcium signals with neuronal spike discharge

In order to determine the correlation between neuronal electroresponsiveness and ΔF/F_0_, we performed WCRs with 200 μ M Fura-2 in the patch-pipette from GrCs and PCs (Figures 4, 5). By using intracellular loading, the recoded cell was unequivocally identified and the electrical and optical signals could be recorded simultaneously.

GrCs (Figure 4A) showed typical passive (high input resistance 1.9 ± 0.3 GΩ, low membrane capacitance 4.2 ± 0.1 pF, *n* = 5) and active electroresponsive properties (D'Angelo et al., 1995; Armano et al., 2000). Injection of depolarizing current pulses generated repetitive spike discharge associated with ΔF/F_0_ of over 10%. The calcium signal increased during spike discharge, continued its growth for tens of ms (78.2 ± 34.5 ms; *n* = 4, *p* = 0.05), reached the peak and then slowly decayed over hundreds of ms (364.2 ± 61 ms; *n* = 4, *p* = 0.05). The relationship between ΔF/F_0_ and the number of GrC spikes was evaluated during repetitive mossy fiber stimulation. The GrCs generated a variable number of spikes probably reflecting quantal variability in the neurotransmission process (Sola et al., 2004). There emerged a quasi-linear correlation of the number of GrC spikes with ΔF/F_0_ peak (*R*^2^ = 0.85; *p* < 0.05; *n* = 5) and time-to-peak (*R*^2^ = 0.94; *p* < 0.01; *n* = 5), as well as of the depolarization area with ΔF/F_0_ area (*R*^2^ = 0.98; *p* < 0.001; *n* = 5) (Figures 4B–E).

PCs (Figure 5) were found to be spontaneously discharging (22.8 ± 2.5 Hz; *n* = 4; *p* < 0.05) and their activity was analyzed while injecting negative currents sufficient to maintain the membrane potential at −70 mV. PC responses to mossy fiber bundle stimulation consisted of either simple spikes or complex spikes. The *simple spikes* were organized in brief spike bursts and longer discharges with variable adaptation (Ojakangas and Ebner, 1992), probably involving variable spike transmission from the GrCs. The ΔF/F_0_ related to simple spikes did not usually exceed 2–3% in PCs, and was thus smaller than in GrCs. The *complex spikes* appeared as high-frequency bursts with marked adaptation. The ΔF/F_0_ of complex spikes was significantly larger (>20%) than that of simple spikes and lasted for hundreds of milliseconds after the end of spike discharge. Moreover, the delay of calcium increase with respect to stimulus onset was significantly lower compared to that observed with simple spike responses (3.7 ± 1.4 ms vs. 12.3 ± 3.6 ms respectively, *n* = 4; *p* < 0.01). As for GrCs, there emerged a quasi-linear correlation of the number of PC simple spikes with ΔF/F_0_ peak (*R*^2^ = 0.9; *p* < 0.01; *n* = 4) and time-to-peak (*R*^2^ = 0.56; *p* < 0.01; *n* = 4), as well as of the depolarization area with the ΔF/F_0_ area (*R*^2^ = 0.63; *p* < 0.05; *n* = 4) (Figures 5B–E).

It should be noted that traces taken from neurons that are either bulk-loaded or intracellularly loaded showed similar ΔF/F_0_ kinetics for rise-time (76.3 ± 5.9 ms vs. 81.7 ± 8.1 ms; *n* = 10 GrCs; *p* = 0.92) and decay time (half-decay: 2.7 ± 0.2 s vs. 2.85 ± 0.3 s; *n* = 10 GrCs; *p* = 0.89). Given that the calcium buffering capacity and the fluorescence of the two dyes is the same, this comparison suggests that the concentrations of Fura-2 and Fura-2AM obtained with the two loading procedures were similar.

It should also be noted that the relationships of ΔF/F_0_ peak vs. spike number and ΔF/F_0_ time-to-peak vs. spike number could be extrapolated toward a single spike, which corresponded to a detectable somatic calcium signal in several cases. From the slope of the ΔF/F_0_ vs. time-to peak relationship it was possible to infer the number of GrC spikes (two-tails *t*-test; *p* = 0.82) and PC spikes (two-tails *t*-test; *p* = 0.99) (Figure 4E). The deviation of the predicted from actual spike number was negligible for GrCs (< ±1 spike) but became evident for PCs when the number of spikes increased to more than five. Therefore, prediction of the number of spikes from ΔF/F_0_ time-to-peak was more reliable for GrCs than for PCs.

### Correlation of calcium signals with the number of input stimuli in the cerebellar network

Mossy fiber bursts composed of different numbers of stimuli (1–10) of identical intensity were used to evaluate the sensitivity of ΔF/F_0_ to the input pattern (Figure 6). The responses were recorded from the GrCs soma and showed a direct relationship between the number of mossy fiber impulses and ΔF/F_0_, which could be fitted with a growing exponential function (*R*^2^ = 0.94; *p* < 0.01). In some cells, signals related to single stimuli were clearly detectable, while in others a step marked the beginning of responses at around 4–5 stimuli. A similar direct relationship could be observed when correlating the number of mossy fiber inputs and ΔF/F_0_ in PCs, which could be fitted with a linear function with 0-intercept (*R*^2^ = 0.81; *p* < 0.05). In this case, the spikes generated by GrCs had to travel through the ascending axon (parallel fibers are severed in sagittal slices) and then activate the PCs. Therefore, the stimuli propagated through the entire network and the intensity of neuronal responses at different network levels was correlated with the number of mossy fiber impulses.

### Organization of activity in multiple cerebellar cortical neurons

**Figure 7:**
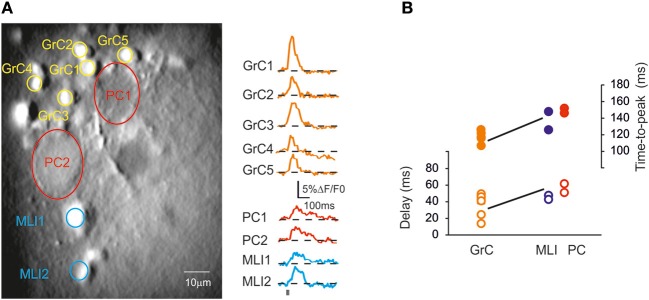
**Cerebellar circuit responses I: neuronal activation sequence. (A)** High magnification (40×) image of a cerebellar slice bulk-loaded with Fura-2AM, in which different cell types can be distinguished. Traces on the right show the signal generated by GrCs, PCs, and MLIs following stimulation of the mossy fiber bundle (10 pulses at 50 Hz). Note the different kinetics and peak amplitude of responses in the different cerebellar neurons. **(B)** The plot shows the time-to-peak (filled circles) and the latency (open circles) of ΔF/F_0_ transients in GrCs, PCs, and MLIs. Note that GrCs activate first and are followed by MLIs and PCs.

Given the possibility of resolving multiple neuronal responses, SLM-2PM recordings were used to characterize the fundamental properties of cerebellar circuit activation. Figure 7 shows several GrCs, PCs and MLIs in an SLM-2PM experiment. The delay from mossy fiber activation was estimated by intercepting the rising phase of the ΔF/F_0_ response at 20% of peak amplitude. Following mossy fiber stimulation, the GrCs were activated first (35 ± 6.1 ms; *n* = 6) followed by the MLIs (45.5 ± 1.5 ms; *n* = 6) and PCs (57.5 ± 2.6 ms; *n* = 6). Thus, MLI excitation was detected 9.8 ± 3.6 ms (*n* = 6) and PC excitation was detected 13.6 ± 5.8 ms (*n* = 6) after GrC excitation.

**Figure 8:**
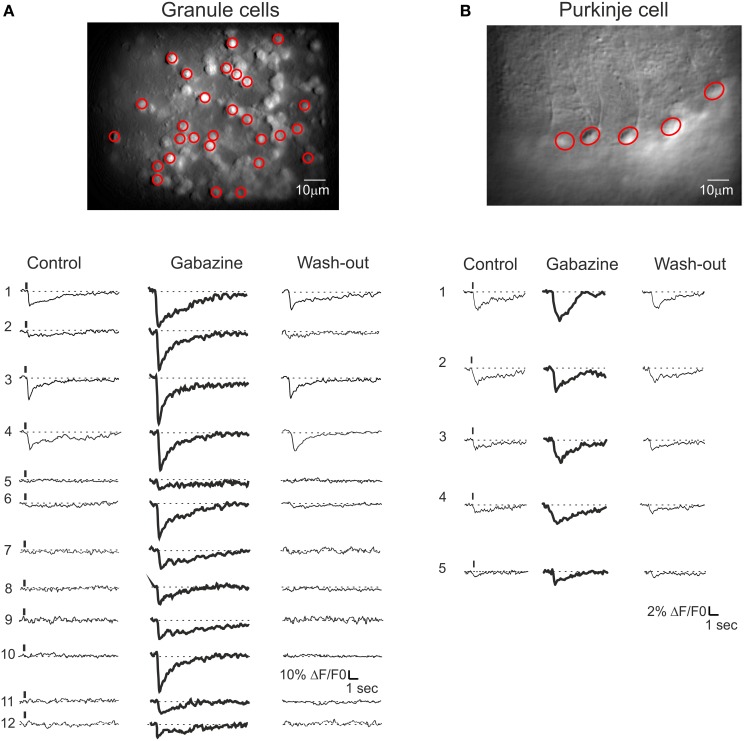
**Cerebellar circuit responses II: multi-neuronal activity patterns**. Activation of the GrC and PC populations ina cerebellar slice bulk-loaded with Fura-2AM. All data were taken from the same experiment but the field of view had to be changed in order to cover the granular layer and molecular layer subfields. The calcium responses were elicited by 10 pulse- 50 Hz mossy fiber burst delivered at 0.1 Hz. **(A)** Granular layer. *Top*, high magnification (40×) image showing fluorescent GrCs (circled in red are those recorded with the SLM-2PM). *Bottom*, example traces showing the responses of GrCs (those circled in red in the top image) to mossy fiber bursts. In control (left), only three GrCs (traces 1, 3, 4) respond to synaptic activation. After application of 20 μ M gabazine, all GrCs respond to synaptic activation and traces 1, 3, 4 are remarkably increased. Finally, after wash-out (right), all GrC responses revert back to control conditions. **(B)** Molecular layer. *Top*, high magnification (40×) image showing fluorescent PCs. *Bottom*, traces showing the responses of PCs (those circled in red in the top image) to mossy fiber bursts. In control (left), only four PCs (traces 1, 2, 3, 4) respond to synaptic activation. After application of 20 μ M gabazine, all PCs respond to synaptic activation and traces 1, 2, 3, 4 are significantly increased. Finally, after wash-out (right), all PC responses revert back to control conditions.

In different experiments (Figures 8A,B), after control recordings, the GABA-A receptor blocker gabazine was perfused. Both the amplitude and the percentage of active GrCs cells and PCs increased after gabazine (cf. Figure 3). Some neurons were inactive in the control condition but their activity appeared with gabazine, attesting to the strong impact of inhibition on the circuit. During washout, all neuronal activities returned to the initial levels, demonstrating full reversibility of drug action and stability of the recordings.

**Figure 9:**
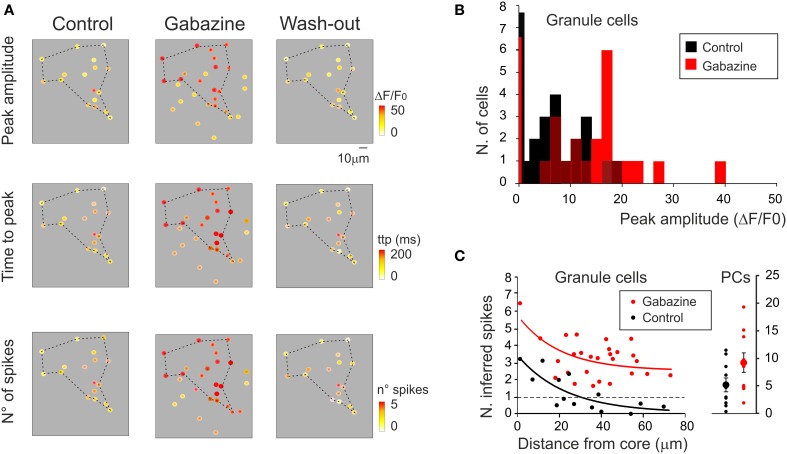
**Cerebellar circuit responses III: predictions of neuronal firing. (A)** Activity maps of the granular layer (same recordings as in Figure 8A) showing the peak intensity of neuronal responses on a color scale. The location of colored dots corresponds to that of neurons in the slice. The panels show that both the activation intensity (upper panels), the time to peak of the response (middle panels) and the inferred number of emitted spikes (lower panels) increase remarkably applying gabazine and revert to control conditions after wash-out. **(B)** The histogram shows the probability of occurrence of response amplitudes in the cell population analyzed in **(A)**. Note the shift of the distribution toward larger amplitude after gabazine application. **(C)** The plot (on the left) shows the relationship between the number of GrCs spikes (inferred from time-to-peak values of the ΔF/F_0_ transient) elicited by mossy fiber stimulation and the distance from center of the responding area. The raster plot (on the right) shows the number of PC spikes (inferred from time to peak values of ΔF/F_0_ transient) elicited by mossy fiber stimulation. Data are taken from the same experiment shown in Figure 8 and in the rest of this figure (control, black; gabazine, red). The number of spikes (*n*) was inferred using the equation *n* = m ^*^ ttp + q, where *ttp* is the time-to-peak of the ΔF/F_0_ transient elicited by mossy fiber stimulation and *m* and *q* are the slope and intercept of the average spike/ttp relationship reported in Figures 4E, 5E. For GrCs *m* = 39.2 and *q* = 56.9; for PCs *m* = 5.8 and *q* = 75.6.

Activity maps were reconstructed (Figure 9A) showing that, with gabazine, the density of active cells and their responses were enhanced (Figure 9B). Moreover, with gabazine, the area covered by responding GrCs increased (+86.3 ± 15.5% *n* = 6; *p* < 0.01), according to the lateral inhibition reported in the granular layer (Mapelli and D'Angelo, 2007). By exploiting the correlation between time-to-peak of calcium transients and spike number (see Figures 4E, 5E), a 2D spike-emission profile was obtained (Figures 9A,C). In response to a mossy fiber burst, the GrCs emitted a limited number of spikes decreasing from center to surround. The 1-spike intercept fell at 29.4 μm indicating an active core of 58.8 μm diameter (cf. Mapelli and D'Angelo, 2007; Mapelli et al., 2010a,b), and the number of spikes increased with gabazine, which caused all the cells to make at least one spike, and some up to six spikes (Figures 9A–C).

**Figure 10:**
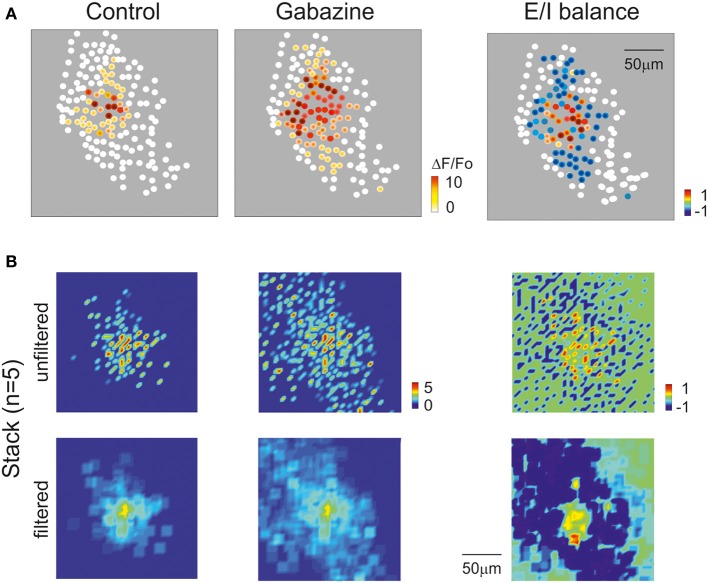
**Cerebellar circuit responses IV: reconstruction of center-surround structure. (A)** Activity maps of the granular layer with 20× magnification showing the peak intensity of neuronal responses on a color scale. The location of colored dots corresponds to that of neurons in the slice. The left most and mid panels show that the activation intensity, number of active cells and extension of activated area increase remarkably by applying gabazine. The E/I balance plot on the right shows that excitation prevails in the core, while inhibition exceeds excitation in the surround. **(B)** Stacks of results obtained from 5 experiments after data normalization. The panels show on color-scale the average response pattern in control, after gabazine and the E/I balance. The plots are shown both before and after spatial filtering. Note in the filtered E/I balance plot the effective reconstruction of the center-surround structure.

In order to test whether the 2PM-SLM data could effectively demonstrate the generation of a center-surround structure, a set of recordings was performed using lower magnification (20×; Figure 10A), in which the field of view was significantly larger than the active area and the contour of excitation was defined. Mossy fiber bursts activated a spot of active GrCs, whose intensity rapidly decayed toward the edge of the spot (*n* = 5). After gabazine perfusion, the number of responding neurons increased, the active area became larger and the intensity of activation in the center of the spot became stronger (cf. Figures 3, 8, 9). The combined effect of inhibition in regulating granular layer activity was obtained by generating “E/I” maps (see methods), where E/I = (*E*_norm_ − *I*_norm_)/*E*_norm_ (Figure 10A; see Methods for details). Obviously, no response could be observed if excitatory fibers did not reach a neuron, even after gabazine perfusion. The map therefore represented the E/I balance in the area activated by excitatory fibers. This map showed that the excitation core was surrounded by a large and deep area of inhibition. The 5 experiments carried out with the 20× objective and analyzed as in Figure 10A were superimposed to obtain a stack image (see methods), which was subsequently filtered (Figure 10B). The average E/I map showed a clear center-surround structure with diameter and active core of 40–60 μm diameter, corresponding to that estimated in Figure 9 by exploiting the relationship between calcium signal and number of activated spikes.

## Discussion

This paper shows that scanless two-photon microscope technologies can be used to perform functional analysis of the entire microcircuit of the cerebellar cortex with single-cell resolution. Up to tens of GrCs and numerous PCs and MLIs could be identified and analyzed simultaneously with calcium indicators. In this study, it proved possible, for the first time, to resolve multiple GrC activities, showing the changes in spike discharge under the control of circuit inhibition. The findings support the center-surround and time-window hypothesis of granular layer activation at the single-cell level.

### Scanless two-photon microscopy in acute cerebellar slices

The SLM-2PM technology developed in this work was based on previous observations showing that two-photon illumination can be used to excite fluorescent calcium dyes and that arbitrary light patterns can be generated using a spatial light modulator (SLM) (Yuste, 2005; Nikolenko et al., 2008; Papagiakoumou et al., 2010; Peterka et al., 2011; Grienberger and Konnerth, 2012). The SLM-2PM, by parking the laser on selected neurons, overcame the limitations imposed by serial point scanning in AOD technologies (Otsu et al., 2008; Sacconi et al., 2008; Yan et al., 2012). Indeed, with AODs, laser beam repositioning allows serial collection of signals from multiple spots at the expense of illumination time and efficiency, making it essential to achieve a critical balance between laser intensity, S/N ratio and number of acquired neurons (Salomé et al., 2006; Duemani Reddy et al., 2008). Using Fura-2AM, the SLM-2PM allowed uninterrupted and stable recordings of multiple neuronal activities. The theoretical limit to the number of recorded neurons was set by the objective(20 × or 40×) determining the field of view and the resolution of neuronal details, the laser power at the specific illumination wavelength (4 W at 800 nm), and the resolution of the CMOS camera. The limit in the present configuration actually derived from the field of view, which suggests that the SLM-2PM has great potential for applications in microcircuit functional investigations. It can be expected that the development of 3D illumination patterns will allow excitation of neurons on different focal planes (e.g., see Kirkby et al., 2010; Fernández-Alfonso et al., 2013, thereby improving the investigation of microcircuit computational and pharmacological properties.

### Identification of neurons and neuronal responses

Cerebellar network activity was elicited by stimulation of the mossy fibers and the neuronal origin of ΔF/F_0_ signals was determined by generating high-resolution confocal images that allowed direct visual matching between fluorescent signals and neuronal elements, by correlating ΔF/F_0_ with the electrical response in WCRs, by correlating ΔF/F_0_ with the neuronal input and output patterns, by evaluating the effect of synaptic receptor antagonists, and by assessing cerebellar cortical network activation as a whole.

In the granular layer, while most neurons are GrCs, it cannot be excluded that some of the recorded signals originated from presynaptic terminals (e.g., see Figure 1C) or other neurons, such as Golgi cells and Lugaro cells (since we recorded from vermis lobules V-VI-VII, the presence of UBCs was unlikely). We were unable to clearly identify Golgi cells and Lugaro cells under light microscopy or with confocal reconstruction in our samples. Since the probability of finding these neurons is relatively low [1 GoC every 430 GrCs (Korbo et al., 1993), 1 Lugaro cell/15 PCs (Dieudonné and Dumoulin, 2000)], it is unlikely that they contributed significantly to signal generation in our recordings. It should also be noted that a contribution of calcium signals generated by glial cells (Hoogland et al., 2009; Hoogland and Kuhn, 2010) cannot be completely ruled out. However, such a contribution is unlikely, given that the typical calcium transients in astrocytes and glial cells show slower kinetics and larger latencies compared with those of neuronal signals and glial calcium waves usually fluctuate independently of synaptic inputs (Hoogland et al., 2009; Hoogland and Kuhn, 2010).

### The nature of neuronal calcium signals

GrC calcium responses were recorded from soma (see also Eilers et al., 1995; Ozden et al., 2012) and showed kinetics similar to those reported previously (rise time >200 ms and decay half-width 2.3 s; see Gall et al., 2005). These calcium signals are likely related to voltage-dependent calcium channels (VDCCs) and calcium-induced calcium release (CICR) in the soma and not to NMDA receptors, which are located in the dendrites. Thus, the calcium signal reduction caused by NMDA receptor blockers should reflect a decrease in membrane depolarization and, secondarily, of calcium entry through VDCCs and CICR. Somatic calcium changes in GrCs were also markedly enhanced by blocking inhibitory activity. The application of the GABA-A receptor blocker gabazine increased the peak amplitude and the number of active neurons, suggesting that some neurons were activated by mossy fibers but initially failed to reach the threshold for signal detection. In this case, too, control of somatic calcium must have been mediated by membrane potential regulation, since GABAergic inhibition generated by Golgi cells is known to limit the amplitude of EPSPs and the number of spikes in GrCs (Armano et al., 2000; Mitchell and Silver, 2003). Given the high-frequency response of the recording system, calcium signal kinetics were probably determined by intracellular calcium dynamics. The similarity of calcium kinetics and ΔF/F_0_ peak signals with Fura-2 and Fura-2AM suggests that the dye concentration was similar in the two cases. Moreover, the low concentration of the dye injected intracellularly is known to have little impact on GrC calcium kinetics (Gall et al., 2003, 2005).

### SLM-2PM analysis of network activation

Both in GrCs cells and in PCs, combined patch-clamp and imaging recordings showed a direct correlation between the number of emitted spikes and the amplitude, area and time-to-peak of the calcium signal. This probably reflected the fact that both in GrCs (D'Angelo et al., 1998, 2001) and in PCs (Lev-Ram et al., 1992; Usowicz et al., 1992; Gruol et al., 2010) VDCCs mostly open during the spike upstroke. In addition, the calcium signal was sensitive to the number of input spikes. Therefore, the SLM-2PM revealed the effect of spike bursts in the main cerebellar cortical neurons and gave indications about their input-output relationship. In PCs it was also possible to distinguish responses generated by both simple spikes and complex spikes. With complex spikes the signal was larger than with simple spikes, possibly because of large calcium influx caused by the high frequency burst and sustained, during the depolarizing wave, by P-type VDCCs (Usowicz et al., 1992; Mark et al., 2011). Thus, the localization and number of active cells was determined from image reconstruction, the nature of spikes (e.g., simple vs. complex spikes) could be related to their amplitude, the number of emitted spikes could be extrapolated from time-to-peak of the calcium response, and the rise time was indicative of the sequential activation of neurons in the network. Finally, the responses of multiple single neurons could be reconnected to the ensemble activation properties reported in MEA and VSD recordings (Mapelli and D'Angelo, 2007; Mapelli et al., 2010a,b). Actually, the superposition of responses centered on the point of maximum activation gave rise to a geometrically organized center-surround structure (see Figure 10).

### Reconstructing circuit functioning from multiple single-neuron activities

By exploiting the correlation between time-to-peak of calcium transients and spike number (see Figures 4E, 5E), a spatiotemporal spike emission profile could be obtained for both GrCs and PCs. In response to a mossy fiber burst, it was possible to estimate from calcium signals that the GrCs emitted a limited number of spikes (1–2) in an active core of 58.8 μm diameter. The number of emitted spikes decreased markedly from center to surround, where several responses were probably subthreshold. The number of spikes increased with gabazine, which caused all the cells to generate at least one spike and some up to six spikes. This is of particular relevance since many theories have been developed about the mechanisms of granular layer functioning but experimental evidence is limited. The behavior of the granular layer has been predicted by theoretical models (Marr, 1969), inferred by genetic mutations (Galliano et al., 2013) and tested with experimental measurements of collective neuronal responses (Hartmann and Bower, 1998; Mapelli and D'Angelo, 2007; Mapelli et al., 2010a,b). Detailed circuit models accounting for granular layer structure and function predicted the distribution of active neurons and their firing pattern (Solinas et al., 2010; Diwakar et al., 2011; Garrido et al., 2013). Here, by comparing SLM-2PM maps, we show, for the first time, that this prediction is accurate and that granular layer activation actually occurs in ~50 μm spots with stronger cell activation in the center than in the surround under control of synaptic inhibition (cf. Figure 10 in D'Angelo et al., 2013), that spike emission from GrCs consists of a short 1–2 spike bursts limited by synaptic inhibition and extended by NMDA receptor activation (cf. D'Angelo and De Zeeuw, 2009).

The sparse activation of GrCs was probably a reflection of the combinatorial arrangement of mossy fiber contacts causing a low percentage of GrCs to cross the threshold for firing (Eccles et al., 1967; Marr, 1969; Ito, 1984). Since Fura-2AMwas applied by bath incubation, sparse responses were probably not due to inhomogeneous cell loading. Moreover, all loaded neurons responded to mossy fiber stimulation, suggesting that these neurons were functionally connected. Therefore, it should be possible to convert this pattern of GrC activation into classical center-surround structures by averaging numerous activity spots, as previously done in acute cerebellar slices (Mapelli and D'Angelo, 2007; Mapelli et al., 2010a), *in vivo* (Roggeri et al., 2008; Diwakar et al., 2011) and in computational models (Solinas et al., 2010).

The delays estimated for granular and molecular layer excitation were obtained by thresholding the calcium signal and are therefore dependent on calcium dynamics rather than just on the time required for neuronal excitation. Once the delay to activate GrCs was factored out, the time required to observe calcium changes in the PC layer was in the order of 10 ms, compatible with known conduction, transmission and excitation times in experiments and computational models (Santamaria et al., 2007; Walter et al., 2009; De Zeeuw et al., 2011). Time-to-peak analysis showed that PC spike number changed from about five spikes in control to about 10 spikes after blocking synaptic inhibition. PCs *in vivo* may fire just 1–2 spikes before being inhibited, therefore five spikes is a number that may reflect inhibitory efficiency in the molecular layer of sagittal slices, in which longitudinal MLI axons are severed. Moreover, the increased PC response after gabazine probably reflected, in part, an increased number of GrC spikes (cf. Figure 6).

## Conclusions

These results demonstrate that use of Fura-2AM calcium imaging through an innovative 2PM-SLM technology makes it possible to record signals precisely correlated with spiking activity generated by multiple cerebellar neurons in acute slice preparations. The 2PM-SLM recordings can provide relevant data for evaluating spatio-temporal firing patterns (De Zeeuw et al., 2011) and testing theoretical predictions such as sparse activation (Marr, 1969), center-surround and time-windowing (Mapelli and D'Angelo, 2007; D'Angelo and De Zeeuw, 2009; Solinas et al., 2010). The use of objectives with a large field of view allows recording of activity from both the granular and the molecular layer. From a broader perspective, these results show that calcium imaging with SLM-2PM technology can be used to monitor the activity of entire neuronal microcircuits at single-cell resolution.

### Conflict of interest statement

The authors declare that the research was conducted in the absence of any commercial or financial relationships that could be construed as a potential conflict of interest.
